# The Efficacy of Red Ginseng in Type 1 and Type 2 Diabetes in Animals

**DOI:** 10.1155/2013/593181

**Published:** 2013-11-11

**Authors:** Bin Na Hong, Min Gun Ji, Tong Ho Kang

**Affiliations:** ^1^Department of Audiology, Nambu University, Gwangju 506-824, Republic of Korea; ^2^Department of Oriental Medicinal Materials & Processing, College of Life Sciences, Kyung Hee University, Global Campus, Gyeonggi 446-701, Republic of Korea; ^3^Graduate School of Biotechnology, Kyung Hee University, Global Campus, Gyeonggi 446-701, Republic of Korea

## Abstract

Diabetes mellitus (DM) is one of the most modern chronic metabolic diseases in the world. Moreover, DM is one of the major causes of modern neurological diseases. In the present study, the therapeutic actions of Korean red ginseng were evaluated in type 1 and type 2 diabetic mouse models using auditory electrophysiological measurement. The comprehensive results from auditory brainstem response (ABR), auditory middle latency response (AMLR), and transient evoked otoacoustic emission (TEOAE) demonstrate auditory functional damage caused by type 1 or 2 DM. Korean red ginseng improved the hearing threshold shift, delayed latencies and signal intensity decrease in type 2 diabetic mice. Type 1 diabetic mice showed a partial improvement in decreasing amplitude and signal intensity, not significantly. We suggest that the Korean red ginseng has a more potent efficacy in hearing loss in insulin resistance type 2 diabetes than in type 1 diabetes.

## 1. Introduction

Diabetes mellitus (DM) is one of the most common modern chronic metabolic diseases in the world. Moreover, DM is a cause of modern chronic neurological disease. Recently, many studies have reported that hearing impairment could be caused by DM [1]. In our previous study, hearing impairment was found in streptozotocin (STZ-) induced diabetic mouse models as caused by type 1 diabetes. STZ-induced DM may impair the auditory pathway from the peripheral auditory nerve to the midbrain in mouse models [2]. Additionally, chronic hyperglycemia and obesity found in type 2 diabetic mouse models may lead to early sensorineural hearing loss [3]. 

Recently, investigations have revealed an improvement in hearing after natural product treatment in hearing-impaired subjects with diabetic hearing loss [4, 5]. In our previous study, coffee or trigonelline ameliorated the hearing threshold shift and delayed the latency of the auditory evoked potential in diabetic hearing impairment [4]. Moreover, diosgenin was observed to clearly cause improvements in diabetic auditory impairment in the hearing threshold, latencies, and otoacoustic emission of electrophysiologic evaluation [5]. 

Korean red ginseng (RG), as an alternative medicine, has been widely used in the Republic of Korea, Japan, and China and is produced by steaming and drying fresh Korean ginseng (*Panax ginseng* C.A. Meyer). This suggests that a chemical transformation of the active physiological properties of ginseng takes place during the production process [6]. RG and its ginsenosides possess multiple pharmacological actions for treating various diseases and conditions including liver regeneration [7], cerebral ischemia [8], and hypertension [9]. Im et al. reported that Korean red ginseng extract could play an antioxidative role in cisplatin-induced ototoxicity through the inhibition of caspase-3 expression in HEI-OC1, an auditory cell line [10]. We reported on a recovery effect of red ginseng on noise-induced hearing loss in mice [11]. Although the antidiabetes effect of red ginseng has been previously reported [12, 13], no study of red ginseng efficacy in diabetic hearing impairment has been reported previously.

In the present study, the therapeutic actions of red ginseng were evaluated in type 1 and type 2 diabetic mouse models using auditory electrophysiologic measurements. To assess the efficacy of red ginseng in diabetic hearing impairment in type 1 and type 2 diabetes, we performed auditory function evaluations as follows: otoacoustic emission (OAE) for the measurement of cochlear functions, auditory brainstem response (ABR) for the measurement of peripheral auditory functions, and auditory middle latency response (AMLR) for the measurement of central auditory functions. 

## 2. Materials and Methods

### 2.1. Korean Red Ginseng Extract

RG extract was obtained from the Korea Ginseng Corporation (Taejon, Republic of Korea). Korean red ginseng extract (crude saponin 70 mg/g, solid component 60%, or more) contained Rb1 (0.46%), Rb2 (0.23%), Rc (0.28%), Rd (0.09%), Re (0.12%), Rf (0.10%), Rg1 (0.07%), Rg2 (0.14%), Rg3 (0.12%), Rh1 (0.10%), and other minor ginsenosides. 

### 2.2. Animal

All of the experimental procedures were performed in accordance with the Principles of Laboratory Animal Care (NIH publication, no. 80-23, revised 1996) and the Animal Care and Use Guidelines of Nambu University, Republic of Korea. Eight-week-old adult male ICR mice were used in the type 1 diabetic mice model, nondiabetic ICR mice were used as the control, and eight-week-old adult male *Lepr* ^(+/+)^ *C57BL/KsJ (dbdb*) mice were used as the type 2 diabetic mice model. Male *Lepr* ^(+/−)^ *C57BL/KsJ (dbh)* littermates were followed concurrently and served as the appropriate control (Jung-Ang Lab Animal, Seoul, Republic of Korea) in this study. The mice were housed individually with a 12 h/12 h light/dark cycle with food and water *ad libitum*.

### 2.3. Induction of Diabetes Mellitus

To induce the type 1 diabetic mice, the ICR mice were injected streptozotocin (STZ) dose of 120 mg/kg. Seven days later, mouse blood glucose values were determined to confirm DM induction. 

### 2.4. Treatment of Red Ginseng

The experimental mice were divided into eight groups (*n* = 10/group). The nondiabetic ICR mice (T1-Con) and the STZ-induced mice (T1-DM) were treated orally once daily with 0.5 mL of distilled water. STZ-induced mice were treated orally once daily with red ginseng 100 mg/kg (T1-R100) and red ginseng 200 mg/kg (T1-R200). The *dbh* mice (T2-Con) and the *dbdb* mice (T2-DM) were treated orally once daily with 0.5 mL of distilled water. The *dbdb* mice were treated orally once daily with red ginseng 100 mg/kg (T2-R100) and red ginseng 200 mg/kg (T2-R200). Solutions containing red ginseng in distilled water were prepared daily immediately prior to treatment. Red ginseng treatments were performed once daily for 8 weeks.

### 2.5. Blood Glucose Level Measurement

Glucose was determined in blood samples obtained by pricking the tail of mice and using a strip-operated blood glucose sensor (ONETOUCH Ultra, Inverness Medical Ltd., UK) under the nonfasting status. Type 1 diabetic mice showing ≥300 mg/dL of blood glucose levels at one week after STZ injection were used.

### 2.6. Auditory Electrophysiologic Evaluation Preparation

All the groups underwent peripheral and central auditory function tests at 8 weeks after red ginseng treatment. The auditory function tests were performed with the mice under anesthesia after an i.m. administration of xylazine (0.43 mg/kg) and ketamine (4.57 mg/kg). The rectal temperature was maintained at 37°C ± 0.5°C using a heating lamp at the time of testing. 

For the auditory electrophysiologic test, two-channel recordings (GSI Audera, Viasys Healthcare Inc., USA) were obtained through needle electrodes inserted s.c. at the vertex. Reference electrodes were placed below the pinnas of the left and the right ears, and a ground electrode was inserted into the shoulder. Electrode impedance was in the range of 2 kΩ to less than 5 kΩ for the electrode pairs. 

### 2.7. Auditory Brainstem Response (ABR) Evaluation

For the ABR recordings, alternating clicks (0.1 ms duration) and 4 and 8 kHz tone bursts (TBs) (rise-plateau-fall; 2-1-2 cycles) were delivered through earphones (Etymotic ER-3A) at a rate of 20.1 stimuli/s. Physiological filters were set to pass electrical activity between 100 and 3000 Hz. Monaural responses were recorded for each mouse and averaged in a 10.24 ms time window. One thousand sweeps were collected. To determine the thresholds in ABR recordings, the clicks were reduced in 10 dB steps. When no response was detected, the level increased in 5 dB steps until a response was determined. ABR parameters were evaluated based on the hearing thresholds and interpeak latencies of waves I–IV at a peak sound pressure level (pSPL) of 90 dB.

### 2.8. Auditory Middle Latency Response (AMLR) Evaluation

For the AMLR measurements, rarefaction clicks (0.1 ms duration) were delivered through earphones at a rate of 9.1 stimuli/s. Filters were set to pass activity between 10 and 250 Hz. An average of 250 sweeps was determined in a 70 ms time window. The parameters of AMLR were evaluated with absolute latencies of wave Pa at a pSPL of 90 dB.

### 2.9. Transient Evoked Otoacoustic Emission (TEOAE) Evaluation

Cochlear function was determined based on transient evoked otoacoustic emissions (TEOAEs) using ILO v6 (Otodynamics, Hatfield, Hertfordshire, UK). TEOAEs were evoked by 80-*μ*s clicks of 90-dB SPL intensity, with a masking noise in the opposite ear, according to the standard nonlinear ILO protocol. TEOAE responses were evaluated in the frequency domain (FFT) by estimating the signal-to-noise ratios (SNRs) at 2 and 3 kHz.

### 2.10. Statistical Analysis

Data were analyzed using the Prism 5 Statistical Software package (GraphPad, San Diego, CA, USA). All data are expressed as the mean ± standard error of the mean (SEM). Statistical comparisons between the groups were performed using a paired *t*-test between 0 weeks and 8 weeks in body weights and blood glucose levels and one-way repeated measured ANOVA with Tukey's *post hoc *test in hearing thresholds, latencies, and SNRs. Values of *P* < 0.05, 0.01, and 0.001 were considered statistically significant.

## 3. Results

### 3.1. Body Weights and Blood Glucose Levels

At 0 weeks and 8 weeks after STZ injection, the body weights and blood glucose levels were evaluated in all the groups (Table 1). The body weights of T1-DM decreased slightly at 8 weeks compared to 0 weeks, whereas the body weights of the T1-Con, T1-R100, and T1-R200 groups increased. The body weights of T2-Con increased slightly at 8 weeks compared to 0 weeks, whereas the T2-DM, T2-R100, and T2-R200 groups significantly increased in body weight from 0 weeks. In the T1-DM, T1-R100, and T1-R200 groups, the mean blood glucose levels 0 weeks and 8 weeks were ≥600 mg/dL, which was the upper measurement limit of the blood glucose analyzer. The blood glucose levels were not decreased in the T1-R100, T1-R200, T2-R100, and T2-R200 groups at 8 weeks. These results show that red ginseng did not improve hyperglycemia in the type 1 and 2 diabetic mouse models used in this study.

### 3.2. Hearing Thresholds in ABR

**Figure 1:**
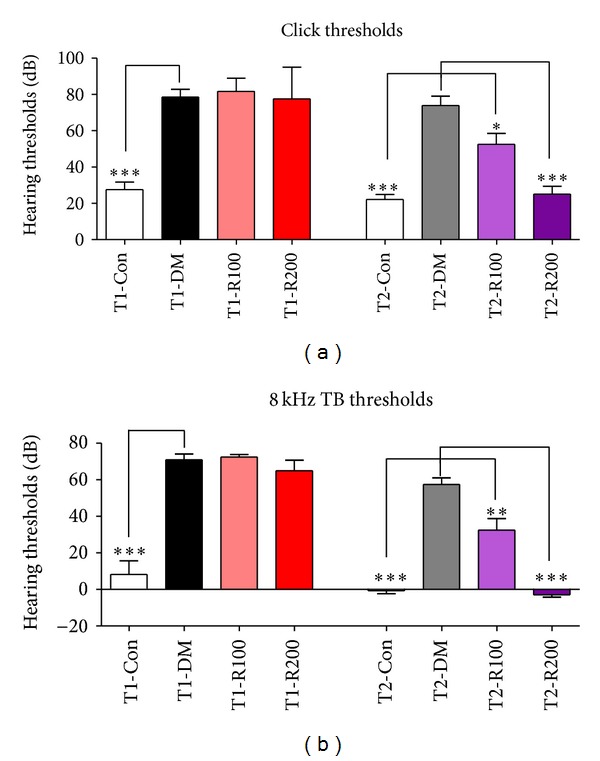
Hearing thresholds of ABR with clicks and 8-kHz TBs in diabetic mice. In this study, the hearing thresholds of ABR after stimulation using clicks (a) and 8-kHz TB stimulations (b) were measured. The data shown indicate the means ± SEM. *P < 0.05, **P < 0.01, and ***P < 0.001 indicate significant differences between the T1-DM and T2-DM groups (Tukey's multiple comparison *post hoc* test).

To assess the ameliorative effects of the hearing threshold shifts in type 1 and 2 diabetic mice, ABR tests were performed at 8 weeks after red ginseng treatments. In the T1-DM and T2-DM groups, the hearing thresholds significantly increased by 61 dB and 61 dB with clicks and by 63 dB and 58 dB with 8-kHz TBs, respectively, compared to the T1-Con and T2-Con groups at 8 weeks. The hearing threshold shifts in the T1-DM and T2-DM groups may induce damages in peripheral nerve function. In the type 1 diabetic mice, the hearing thresholds of the red ginseng treatment groups (T1-R100 and T1-R200) slightly decreased compared to the T1-DM group. In the type 2 diabetic mice, the hearing thresholds of the red ginseng treatment groups (T2-R100 and T2-R200) significantly decreased by 21 dB and 48 dB with clicks and by 25 dB and 60 dB with 8-kHz TBs, respectively, compared to the T2-DM group (Figures 1(a) and 1(b)). These data indicate that red ginseng suppresses the hearing threshold shifts in the type 2 diabetic mouse model and improves auditory nerve functions, whereas no improvement was observed in the type 1 diabetic mouse model.

### 3.3. Peak Wave Latencies in ABR

**Figure 2:**
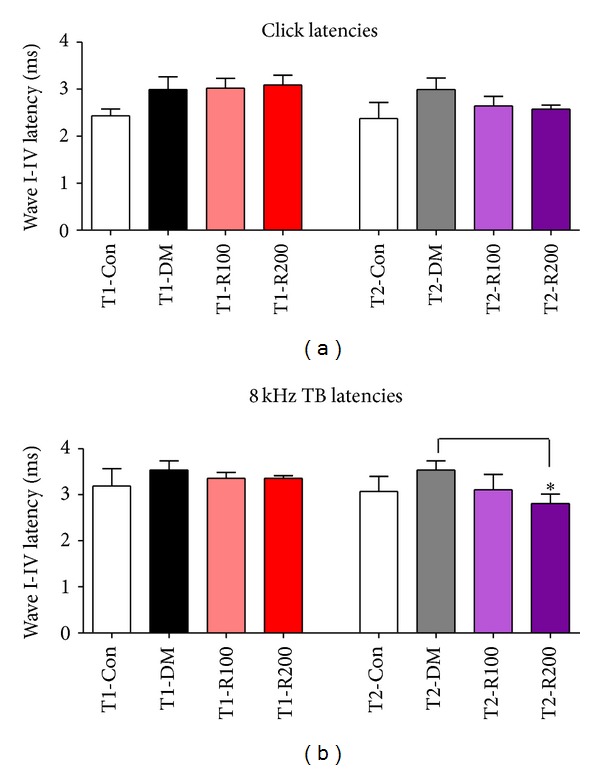
Latencies of ABR with clicks and 8 kHz TBs in diabetic mice. In this study, the wave I–IV latencies of ABR after stimulation using clicks (a) and 8 kHz TB stimulations (b) were measured. The data shown indicate the means ± SEM. *P < 0.05 indicates a significant difference from the T2-DM group (Tukey's multiple comparison *post hoc* test).

To assess the ameliorative effects of auditory nerve damage in type 1 and 2 diabetic mice, ABR tests were performed at 8 weeks after red ginseng treatments. In the T1-DM and T2-DM groups, the peak latencies increased compared to the T1-Con and T2-Con groups at 8 weeks. The latency delay in the T1-DM and T2-DM groups may be damaged for peripheral nerve conductivity. In type 1 diabetic mice, the latencies of the red ginseng treatment groups (T1-R100 and T1-R200) were similar to the T1-DM group. In type 2 diabetic mice, the latencies of the red ginseng treatment group (T2-R200) significantly decreased compared to the T2-DM group (Figures 2(a) and 2(b)). These data indicate that red ginseng suppresses latency delay in the type 2 diabetic mouse model and improves auditory nerve functions. 

### 3.4.  Pa Latency and Amplitude in AMLR

**Figure 3:**
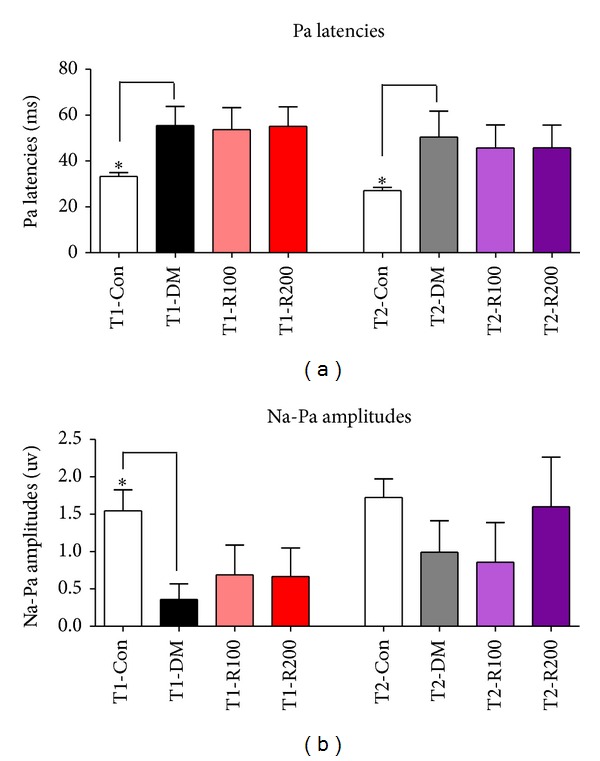
Latencies and amplitudes of AMLR in diabetic mice. In this study, Pa latencies (a) and Na-Pa amplitudes (b) of AMLR after stimulation using clicks was measured. The data shown indicate the means ± SEM. *P < 0.05 indicates significant differences between the T1-DM and T2-DM groups (Tukey's multiple comparison *post hoc* test).

To assess the ameliorative effects of central auditory function damage in type 1 and 2 diabetic mice, AMLR tests were performed at 8 weeks after red ginseng treatments. In the T1-DM and T2-DM groups, Pa latencies in AMLR significantly increased compared to the T1-Con and T2-Con groups. In type 1 diabetic mice, the Pa latencies of the red ginseng treatment groups (T1-R100 and T1-R200) were similar to the T1-DM group. In type 2 diabetic mice, the Pa latencies of the red ginseng treatment groups (T2-R100 and T2-R200) decreased slightly compared to the T2-DM group (Figure 3(a)). In the T1-DM and T2-DM groups, the Na-Pa amplitudes in AMLR significantly decreased compared to the T1-Con and T2-Con groups. In type 1 diabetic mice, the Na-Pa amplitudes of the red ginseng treatment groups (T1-R100 and T1-R200) slightly increased compared to the T1-DM group, but this increase was not significant. In the type 2 diabetic mice, the Na-Pa amplitudes of the red ginseng treatment groups (T2-R200) increased compared to the T2-DM group, but this increase was not significant (Figure 3(b)). These data indicate that red ginseng suppresses latency delay, decreases amplitude in the type 2 diabetic mouse model, and improves the central auditory functions compared to the type 1 diabetic mouse model.

### 3.5. Signal-to-Noise Ratios in TEOAE

**Figure 4:**
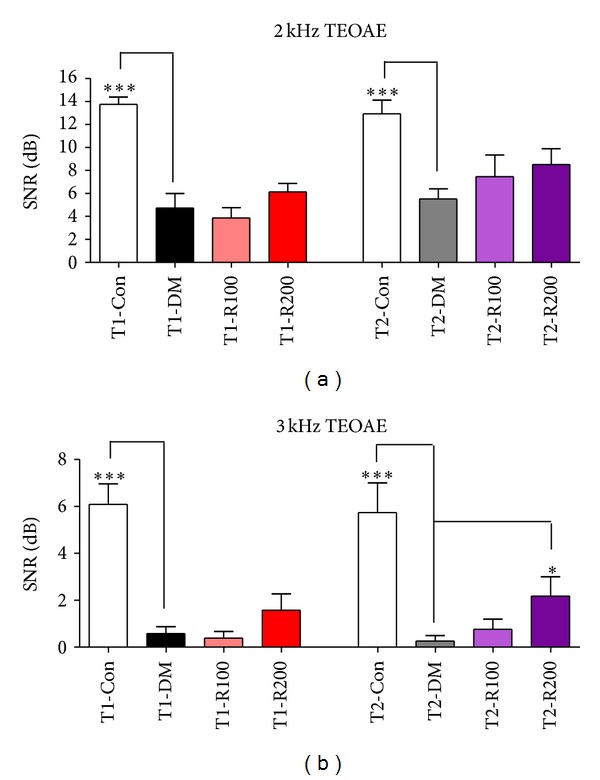
Signal intensities of TEOAEs with 2 and 3 kHz in diabetic mice. In this study, the signal intensity of TEOAEs to noise at 2 kHz (a) and 3 kHz TBs (b) were measured. The data shown indicate the means ± SEM. *P < 0.05 and ***P < 0.001 indicate significant differences between the T1-DM and T2-DM groups (Tukey's multiple comparison *post hoc* test).

To assess the ameliorative effects of outer hair cell damages in type 1 and 2 diabetic mice, TEOAEs with 2 and 3 kHz were performed at 8 weeks after red ginseng treatments. The SNRs of 2 and 3 kHz of TEOAEs in the T1-DM and T2-DM groups significantly decreased at 8 weeks compared to the T1-Con and T2-Con groups. In type 1 diabetic mice, the SNRs of the red ginseng treatment groups (T1-R200) slightly increased compared to the T1-DM group. In type 2 diabetic mice, the SNRs of the red ginseng treatment groups (T2-R100 and T2-R200) increased compared to the T2-DM group (Figures 4(a) and 4(b)). These data indicate that red ginseng suppresses the decrease of SNRs in the type 2 diabetic mouse model and improves outer hair cell function in the cochlea compared to the type 1 diabetic mouse model.

## 4. Discussion

The comprehensive results from ABR, AMLR, and TEOAE demonstrate auditory functional damage caused by type 1 or 2 DM. Red ginseng improved the hearing threshold shift, delayed latencies, and decreased signal intensity in type 2 diabetic mice. Type 1 diabetic mice exhibited a partial improvement in the decrease in amplitude and signal intensity, but these improvements were not significant. The question then remains regarding the reason that there is a significant improvement in auditory function in type 2 diabetes and not type 1 diabetes.

Diabetes mellitus is classified as type 1 or type 2. The metabolic pathologies of type 1 diabetes mellitus (T1-DM) and type 2 diabetes mellitus (T2-DM) are different. T1-DM is caused by the loss of beta cells found in the islets of Langerhans in the pancreas. T2-DM is associated with insulin resistance and relative insulin deficiency [14]. T1-DM has symptoms of hyperglycemia and hypoinsulinemia while T2-DM has symptoms of hyperglycemia and hyperinsulinemia or insulin resistance. Therefore, the neurological diseases associated with T1-DM and T2-DM are different, especially in relation to insulin. 

Fukushima et al. [15] have described DM-associated pathology changes within the cochlea that include thickened vessels of the stria vascularis, atrophy of the stria vascularis, and loss of outer hair cells in humans. Altered auditory function in diabetic animals has been examined in both otoacoustic emissions [16] and auditory brainstem responses, as well as in auditory middle latency responses [2]. Current research indicates that DM may cause hearing impairment, but a firm cause-effect correlation according to DM type has not yet been described [17]. A number of studies have attempted to identify the differences in hearing loss in humans with type 1 and type 2 DM [18–20], but, to date, the location of the lesions and the mechanism of deficit have not been established. Recent studies showed distinct differences in the severity of polyneuropathy in patients with type 1 and type 2 diabetes. Progressive axonal atrophy and loss are more serious in patients with type 1 diabetes, while patients with type 2 diabetes exhibit nodal and paranodal degenerative changes as well as more severe downstream effects on neuroskeletal and adhesive proteins [21].

Korean red ginseng (RG) is believed to contain ingredients that possess a variety of health enhancing effects including an antidiabetic effect [22, 23], enhanced erectile function [24], and cognitive enhancement [25]. Recently, RG has been shown to improve cognitive function related to the auditory pathway using electrophysiologic evaluation [26]. 

Several human studies have reported that the administration of KRG had positive effects on the maintenance of sugar control and insulin resistance in type 2 diabetes mellitus patients [27, 28]. In addition, a previous animal study suggested the potential beneficial effects of KRG on the amelioration of insulin resistance and prevention of T2-DM through the activation of the AMP-activated protein kinase (AMPK) in fat rats [29]. Although the effect of red ginseng on nerve protection has not been reported, the nerve protection efficacy of Rb1, a major ginsenoside of red ginseng, has been reported in many studies [30, 31].

In this study, although red ginseng did not improve hyperglycemia, red ginseng improved diabetic hearing impairments observed in type 2 diabetic mice. We suggest that red ginseng efficacy in the auditory function of type 2 diabetic mice may be related to the improved insulin sensitivity of red ginseng and protection efficacy of the nerve from the major ginsenoside of red ginseng. Additionally, the mouse strain difference of types 1 and 2 diabetic mice in this study or other factors may have been influenced. More studies will be needed in the future.

## 5. Conclusions

In the present study, the therapeutic actions of red ginseng were evaluated in type 1 and type 2 diabetic mouse models using auditory electrophysiological measurement. The comprehensive results from ABR, AMLR, and TEOAE demonstrate auditory functional damage caused by type 1 or 2 DM. Korean red ginseng improved the hearing threshold shift, delayed latencies, and signal intensity decrease in type 2 diabetic mice. Type 1 diabetic mice showed a partial improvement in decreasing amplitude and signal intensity, not significant. We suggest that the Korean red ginseng has a more potent efficacy on hearing loss in insulin resistance type 2 diabetes than in type 1 diabetes.

## Figures and Tables

**Table 1:** Body weights and glucose levels in diabetic mice.

Groups	Body weight (g)		Glucose levels (mg/dL)	
	0 weeks	8 weeks	0 weeks	8 weeks
T1-Con	38.4 ± 1.5	40.8 ± 1.4	136.9 ± 6.8	133.8 ± 6.2
T1-DM	39.6 ± 2.5	38.8 ± 2.8	≥600.00	≥600.00
T1-R100	37.2 ± 2.5	40.0 ± 1.2	≥600.00	≥600.00
T1-R200	37.8 ± 2.0	40.0 ± 3.0	≥600.00	≥600.00
T2-Con	20.5 ± 0.5	23.3 ± 0.5	168.4 ± 0.8	177.8 ± 8.7
T2-DM	42.4 ± 0.9	65.9 ± 1.9***	267.5 ± 19.4	364.5 ± 30.2*
T2-R100	41.7 ± 0.9	65.0 ± 2.3***	310.8 ± 17.5	392.3 ± 15.8*
T2-R200	42.2 ± 1.3	66.9 ± 1.6***	335.6 ± 32.0	364.7 ± 30.7

Nondiabetic ICR mice (T1-Con), STZ-induced diabetic mice as the type 1 diabetic mice model (T1-DM), STZ-induced diabetic mice treated with RG 100 mg/kg (T1-R100), STZ-induced diabetic mice treated with RG 200 mg/kg (T1-R200), *dbh* mice (T2-Con), *dbdb* mice as the type 2 diabetic mice model (T2-DM), *dbdb* mice treated with RG 100 mg/kg (T2-R100), and *dbdb* mice treated with RG 200 mg/kg (T2-R200) groups were evaluated for their body weights and blood glucose levels. The data shown indicate the means ± SEM. *P < 0.05 and ***P < 0.001 indicate significant differences from the values at 0 weeks.
